# Machine-designed biotherapeutics: opportunities, feasibility and advantages of deep learning in computational antibody discovery

**DOI:** 10.1093/bib/bbac267

**Published:** 2022-07-14

**Authors:** Wiktoria Wilman, Sonia Wróbel, Weronika Bielska, Piotr Deszynski, Paweł Dudzic, Igor Jaszczyszyn, Jędrzej Kaniewski, Jakub Młokosiewicz, Anahita Rouyan, Tadeusz Satława, Sandeep Kumar, Victor Greiff, Konrad Krawczyk

**Affiliations:** NaturalAntibody; NaturalAntibody; NaturalAntibody; NaturalAntibody; NaturalAntibody; NaturalAntibody; Warsaw Medical University; NaturalAntibody; NaturalAntibody; NaturalAntibody; NaturalAntibody; Boehringer Ingelheim; University of Oslo and Oslo University Hospital; NaturalAntibody

**Keywords:** antibody, drug discovery, machine learning, deep learning, artificial intelligence, immunoinformatics

## Abstract

Antibodies are versatile molecular binders with an established and growing role as therapeutics. Computational approaches to developing and designing these molecules are being increasingly used to complement traditional lab-based processes. Nowadays, *in silico* methods fill multiple elements of the discovery stage, such as characterizing antibody–antigen interactions and identifying developability liabilities. Recently, computational methods tackling such problems have begun to follow machine learning paradigms, in many cases deep learning specifically. This paradigm shift offers improvements in established areas such as structure or binding prediction and opens up new possibilities such as language-based modeling of antibody repertoires or machine-learning-based generation of novel sequences. In this review, we critically examine the recent developments in (deep) machine learning approaches to therapeutic antibody design with implications for fully computational antibody design.

## Introduction

The number of newly approved antibody-based therapeutics is rapidly increasing. We have already passed the point of 100 Food and Drug Administration approvals with multiple antibodies in clinical trials and patent filing stages [1, 2]. This is reflected in the market size for these molecules, estimated at $130 billion in 2020 and projected to grow to 223 billion by 2025 [3, 4]. Most of the antibodies on the market were developed using costly and time-consuming techniques, chiefly phage display or animal immunization platforms [5, 6]. With the maturity and increasing integration of computational protocols within pharma company pipelines, the time and cost associated with therapeutic antibody development are expected to decrease. This shall hopefully make immunotherapy more affordable to patients and widen the applicability to more disease conditions.

Our previous reviews delineated the computational resources available to antibody engineers [7]. Most of the tools we reported on covered various statistical techniques such as homology modeling for structure prediction and z-scores for humanness annotation. The increasing availability of large-scale data on B-cell receptors [8, 9] and advances in machine learning-based model development [10–12] are significant developments in the computational antibody field within the last few years. Such advancements appear to have contributed to several computational approaches to therapeutic antibody discovery following the deep learning paradigm. This trend not only resulted in employing such methods to tackle well-established problems (e.g. structure prediction) but also created entirely new fields (e.g. generative models for novel antibody design).

In this review, we describe the recent developments in computational antibody engineering, specifically highlighting the novel applications of deep learning. We present the methods that improve the previous state-of-the-art (e.g. structure prediction and humanization) but also introduce novel concepts such as language-motivated embeddings and automated sequence generation. The new paradigm shift towards machine learning—encapsulated by embedding and generative methods—offers a novel way of designing antibody-based therapeutics computationally.

## Encoding antibody, antigen sequence and structure for machine learning applications

Feature engineering is the process of creating new artificial input features from raw data to improve model performance. This process is vital in developing machine learning models that apply to biological data—to draw the connections between sequence and phenotype, one needs to formalize the biological representations [13]. In the context of antibodies, we chiefly distinguish between sequence, structure and graph representations.

**Figure 1:**
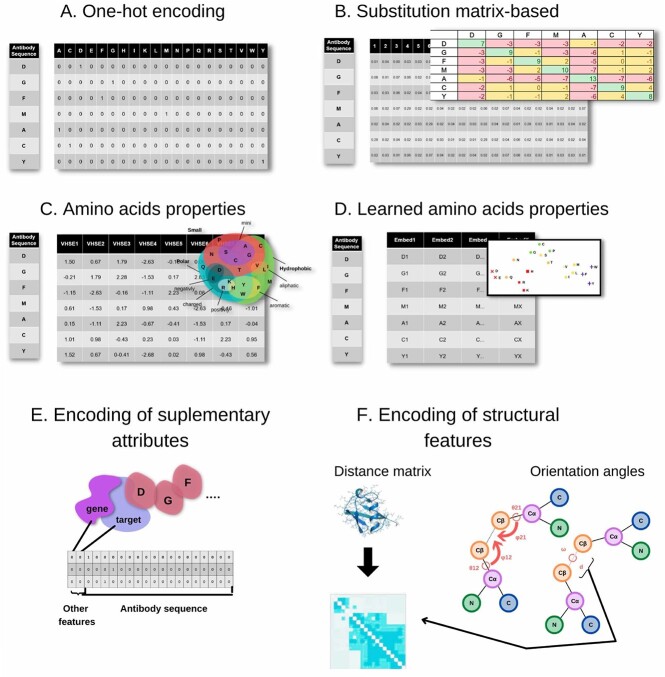
Antibody encoding schemes. (**A**) One-hot encoding. Sparse vector representation for each residue with 1 for amino acid present and 0 s for remaining positions. (**B**) Substitution matrix. Rather than 0/1 as in one-hot encoding, each amino acid present receives a score from the substitution amino acid matrix, e.g. Blosum. (**C**) Amino acid properties. Similarly to substitution-matrix approaches, scores encapsulate knowledge-based properties, such as size, charge, etc. (**D**) Learned amino acid properties. Infer embeddings for each amino acid based on training of the network. (**E**) Encoding of supplementary attributes such as organism, gene, etc., alongside amino acid encoding. (**F**) Encoding of structural features. For invariant representations, structures can be represented by distance matrices or by orientation angles between consecutive amino acids.

One of the most basic approaches to encode antibody sequence information is to apply one-hot-encoding (Figure 1A), where each letter representing residue in the protein chain is replaced by a 20-element vector, with ‘1’ in place for represented amino acid and ‘0’ for others. Such vectors can account for gaps or the start/end of the sequence.

Such basic representation can be extended by replacing 0/1 with encodings reflecting amino acid properties. For this purpose, one can use substitution matrices (e.g. Blosum, Figure 1B) that capture evolutionary relationships. Here each amino acid is encoded as a 20-element vector, in which each element represents a value taken from the substitution matrix. Another option is using an encoding that encapsulates known physicochemical properties of amino acids (e.g. Vectors of Hydrophobic, Steric, and Electronic (VHSE) properties [14], Figure 1C), where the residue representation vector contains values of known hydrophobic, steric and electronic properties. In this approach, it is common to apply dimensionality reduction algorithms (e.g. Principle component analysis (PCA)) to reduce the size of the representation vector.

In contrast to manually adding domain knowledge to encodings, vectorizations for individual amino acids can be also learned together with model parameters in end-to-end learning (Figure 1D) [14]. Such task-specific learned representation yields similar performance compared to other encodings mentioned above, while keeping a smaller vector size. This lower dimensionality may be important in cases of deploying models to devices with limited computing capacity or when operating on large volumes of data, where processing time translates directly to cost.

Amino acid encodings can also be supplemented by additional details (Figure 1E) such as organism, gene and more importantly, positional information. Numbering schemes [15, 16] for antibodies act as an implicit multiple sequence alignment that contextualizes the amino acid residues in their functional positions (e.g. framework, Complementarity Determining Region (CDR)). Amino acid representation can take such positional dependencies into account (e.g. input neuron 68 will correspond to international ImMunoGeneTics information system (IMGT) position 56) but can also be approached by learning alignment-free dependencies [17].

Sequence-based encodings provide an initial layer of information for three-dimensional (3D) structure encodings. Since structural elements are interdependent in 3D space, the machine learning method must either operate within a well-defined frame of reference or remove the variance altogether. One can define a single frame of reference by aligning all the structures together [18] and predicting the X, Y, Z coordinates. Another approach is to make the coordinates insensitive to rotation and translation by operating on invariant features. Such features are the distances between atoms and the orientation angles (Figure 1F) [19].

A special case of structural representation is using graphs. Protein structure and function result from an inter-residual interaction network, which can be abstracted into a graph where amino acid residues are nodes and contacts or interactions between them constitute graph edges. Such representation is denoted as Residue Interaction Network or Protein Contact Network and can be constructed using varying nodes and edges definitions. For example, the Cα or Cβ atoms of a residue can be nodes, and edges are drawn based on the distances between them [20, 21]. It is also possible to construct a network of non-covalent interactions between residues [22]. Here, each amino acid is represented as a graph node, and edges are drawn where noncovalent interaction strength is above an interaction strength threshold. There are also variations, which combine angle with distance information where each edge connecting residues consists of four parts: their positional distance, radial distance, direction encoding and orientation encoding [23].

Alongside input encoding, it is equally important to encode the predicted values suitably. Here, predictions can be divided into categorical and continuous. Examples of categorical predictions include predicting the source of an antibody (e.g. murine or human) from its amino acid sequence [24] or the incidence of amino acids at specific positions in protein sequence [25]. On the other hand, continuous predictions aim to capture values such as aggregation propensity [26] or orientation angles of residues in a structure [27]. Encoding the categorical values typically takes the form of an n-dimensional vector where n is the target number of classes—for instance, in attempting to call an organism based on an antibody’s sequence, one could encode labels as (1,0) for mouse and (0,1) for human. Since predicting continuous functions is naturally challenging, the continuous variables are often bucketed into equally sized intervals. For instance, the prediction of pairwise residue distances in a protein structure is naturally an continuous problem; prediction can take the form of N equally spaced intervals with an upper bound on maximal predicted distance [19].

Although, as described here, multiple ways of encoding sequences and structures exist, these are not exclusively associated with model architectures. For instance, one-hot encoding can be used both to encode heavy chain CDR3 input to a convolutional neural network (CNN) for binding prediction and a heavy chain sequence for coordinate prediction by a ResNet. The choice of architecture is related to the problem that it attempts to address.

## Common network architectures employed for therapeutic antibody issues

Current strategies to tackle antibody problems are shifting towards machine learning in general and deep learning in particular, comprising a specific set of techniques to design and train artificial neural networks. Here we summarize some of the architectures and methods currently used in antibody immunoinformatics.

**Figure 2:**
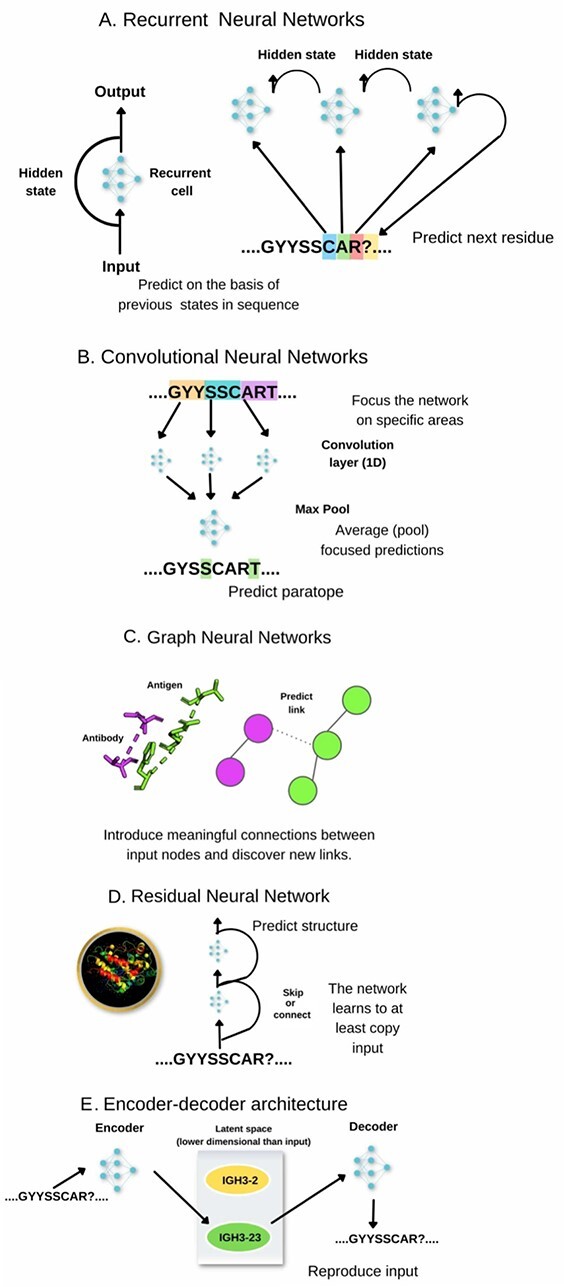
Some common neural network architectures and concepts in the context of some antibody-specific problems. Simplified examples are given to show potential applications on sequence/structural inputs with the networks capable of operating on more complex inputs (e.g. entire variable region sequences rather than just CDR-H3 or more complex molecular descriptors than just atomic coordinates). (**A**) Recurrent networks. Information is read one element at a time, maintaining a hidden state. This architecture is often used for sequence-based input such as CDRs or variable region sequences. (**B**) Convolutional Neural Networks. Predictions are constrained to portions of the input and are then pooled together. Such networks can focus on local patterns and combining them into predictions, making them useful in identifying motifs in sequences or identifying molecular surface features. (**C**) Graph Neural Networks. The abstract linkage between elements in input can be reflected. Such networks can process abstract representations of molecules. (**D**) Residual Neural Networks. Portions of the network can be circumvented, allowing for deeper networks without risking exploding or vanishing gradients. Such networks were used with great success for structure prediction. (**E**) Encoder-Decoder networks. The input is encoded into a latent representation by reducing the dimensionality and attempting to reconstruct the input. The resulting latent representation can reflect intrinsic features of the input, such as gene assignments and propensity towards similar targets.

Recurrent neural networks (RNNs) [28] (Fig. 2A) receive as input an entry in a sequence/time series and a hidden state of the previous recurrent cell. Each step produces an output value and the next hidden state. This kind of architecture allows processing sequences with different lengths. Examples here include the long-short term memory networks [29] or gated recurrent unit [30]. For example, Wollacott et al. have recently applied a bidirectional long short term memory network (LSTM) (analyzing sequence going forward and backward) to understand and predict the organism (nativeness) of an antibody sequence [31].

Standard neural networks may accept data that does not have any internal structure. On the other hand, if our goal is to analyze data that exhibits some non-trivial structure—e.g. our data points are sequences of letters or images composed of pixels—we can design architectures that take advantage of this additional information. Some internal structure allows us to talk about local features, i.e. patterns that can be identified by investigating only a portion of the input. CNNs, Figure 2B identify local features invariant to their global position. In antibodies, this translates into focusing the convolutional layer on consecutive sequence stretches or 3D configurations of atoms in structures, which was employed for structure [18] and binding prediction [32, 33].

Graph Neural Networks (Figure 2C) encapsulate a similar paradigm for encoding structural features. Graph representation focuses on input entities (e.g. residues) and their relationships (e.g. distance<4 Å) rather than on absolute positions, therefore it is insensitive to input rotations. Such a graph model can then be used for link prediction and for structure generation within the antibody–antigen context [23].

ResNet is a type of network that utilizes *skip connections* between layers (Figure 2D). Adding this type of shortcut between layers solves the problem with *vanishing gradients*, allowing for the training of intense networks. The most popular ResNets used for image recognition contain 34, 50, 101 or even 150 layers. Commonly used architectures consist of several stacked blocks, each composed of two or three connected layers with skip connections over them. In the context of antibodies, ResNet was used by NanoNet [11] to predict the structure of heavy chains.

One of the main tasks of machine learning methods is to arrive at an input representation internal to the neural network that allows performing the predictions efficiently. This implicitly makes the network learn the hidden, latent representation that identifies input features and their relationships. Such representation is a model of the distribution of input values. Though each network does it implicitly, one can also train the network to achieve this task on purpose, whence one can control the properties of the latent space. In such encoder–decoder architecture (Figure 2E), the network attempts to encode the input in a lower number of dimensions (encoder) and then reconstruct the original input from it (decoder). Several network architectures attempt that, such as variational autoencoders (VAEs) [34], Generative Adversarial Networks [35] or Transformers [25]. Latent representations can be trained from voluminous unlabeled datasets (e.g. Next generation sequencing (NGS)), and then the models used to train further on much smaller labeled datasets (e.g. paratope prediction) in the transfer learning process.

Many architectures describe above lack explainability in that it is difficult to judge which features contribute to the final predictions, which can be associated with biological interpretation [36]. An important explainability component (among others) that has been applied to antibodies is *attention*. In classical models, input sequences are compressed by an encoder to a fixed size length vector (context), which is further used for prediction or sequence modeling. This representation becomes a bottleneck when the input sequence is long. Attention [37] overcomes this problem by computing the context vector as a weighted average of all intermediate encoder outputs. Attention weights are calculated by applying the softmax function over *attention scores,* which are calculated by a small feedforward network. This mechanism, therefore, allows selecting input elements that contribute more to better predictions and boost performance, especially in sequence-to-sequence tasks. Attention plays a crucial role in the Transformer model [38] and other state-of-the-art networks for text and sequence processing with recent antibody applications [25, 39].

## Old problems, new solutions—novel deep learning applications to traditional computational antibody problems

Computational tools used to facilitate the therapeutic design of antibodies can be divided into two broad categories; ones focused on predicting antibody–antigen interactions [20, 32, 58] and ones focused on the developability properties of antibodies [26, 59]. As a basis for many of such methods, one needs to determine the 3D coordinates of the antibody molecule [60]. Accurate structure predictions can enrich sequence information with molecular features [61], useful for machine learning approaches to binding and developability prediction.

## Embedding antibody 3D space–structure prediction

Prediction of antibody structure has wide-ranging applications in antibody engineering as the molecular shape of the paratope defines the antibody–antigen recognition [61]. Determining proteins’ crystal structure is technically challenging, prompting a wide interest in developing methods for predicting the 3D coordinates from sequence alone [62]. While there are thousands of crystal structures available for fragments of antibodies such as Fabs, Fcs, Fvs, the number of full-length antibody structures amounts to merely six. Moreover, there are no crystal structures of multispecific antibodies available. Until recently, the method of choice for tackling this problem was homology modeling and energy-based methods.

The advent of machine learning in the field recently culminated in a spectacular performance by AlphaFold2 (AF2) at CASP 14 [10, 62]. Protein structure prediction methods such as AF2 derive much information from coevolutionary signals [63, 64]. Because of the specific nature of the antibody problem, methods such as AF2 are not explicitly designed for capturing small structural nuances such as hundreds of millions of available CDRH3 structures [65].

Because of their specific biology, the structural prediction of antibodies has been a separate sub-area of protein structure prediction. However, it has always benefited from the progress in the broader field. As initial protein structure predictions were homology and energy-based, so were some of the first methods tackling this problem [60, 66–69]. The nuance here is that predictions are separated between frameworks (that are structurally conserved) and CDRs, especially the most variable one, the CDRH3 loop. The most recent methods that address antibody-specific CDRH3 predictions are DeepH3 and ABlooper (Table 1).

**Table 1:** Recent examples of machine learning applications in antibodies

**Category**	**Method**	**Problem solved**	**Training input**	**Architecture**	**Training parameters**	**Libraries**	**Availability**	**Paper**
Structure prediction	DeepH3	CDRH3 prediction	1388 structures	Series of 1D and 2D convolutions (3 1D + 25 2D blocks)	30 epochs, batch size 4, 35 h using one NVIDIA Tesla K80 Graphics processing unit (GPU)	PyTorch	link	[27]
	DeepAb	V region structure prediction	118 386 sequences and 1692 structures	A 1D ResNet (1D convolution followed by three 1D ResNet blocks) and the bi-LSTM encoder	60 epochs, batch size 128, NVIDIA K80 GPU requiring 60 h	PyTorch	link	[19]
	AbLooper	CDR Prediction	3438 structures	Five E(n)-equivariant graph neural networks (EGNNs), each one with four layers	NVIDIA Tesla V100 GPU, predict the CDRs for one hundred structures in under five seconds	PyTorch	link	[40]
	NanoNet	Heavy chain prediction	~2000 structures	Two 1D ResNets with input tensor of 140 × 22	batch size of 16 and ~ 130 epochs,10 min on a GeForce RTX 2080 Ti	Keras/TensorFlow	link	[18]^*^
Humanization/Deimmunization	Nativeness LSTM	Learn distribution of amino acids at positions	400 000 sequences	Bidirectional LSTM with dimensionality 64	10 epochs	PyTorch	link	[31]
	Sapiens	Antibody humanization	20 milion heavy chains and 19 milion light chains	RoBERTA transformer, 4 layers, 8 attention heads, 568 857 parameters	700 epochs for heavy chains, 300 epochs for light chains	PyTorch/Fairseq [41]	link	[24]
	hu-Mab	Discriminate between human/mouse sequences	65 million sequences with 13 million non-human ones	Random Forest	n/a	scikit-learn	link	[42]
Binding models	Parapred	Paratope residues prediction	1662 sequences (277 antibody–antigen complexes × 6 Complementarity determining regions each) and tested on the same dataset using 10-fold cross-validation technique	Convolutional and recurrent neural networks	16 epochs, 32 batch size	Keras	link	[43]
	Epitope3d	Conformational epitopes prediction	1351 antibody–antigen structures (covering 40 842 epitope residues) and 180 unbound antigen structures; tested on 20 unbound antigen structures; 45 unbound antigen structures used for external blind test	Supervised learning algorithms: Multi-layer Perceptron, Support Vector Machines, K-Nearest Neighbor, Adaboost, Gaussian processes (GP), Random Forest, Gradient Boost, XGBoost, Extra Trees	N/A	scikit-learn Python	link	[44]
	mmCSM-AB	Prediction of the consequences of multiple point mutations on antibody–antigen binding affinity	1640 mutations with associated changes in binding affinity (905 single missense mutations and 735 modeled reverse mutations); tested on 242 multiple missense mutations with associated changes in binding affinity	Supervised learning algorithms for example: Random Forest, Extra Trees, Gradient Boost, XGBoost, SVM and Gaussian Process	n/a	scikit-learn Python	link	[45]
	Phage display LSTM	Generate novel kynurenine binding sequences from LSTM	959 sequences	LSTM, two layers with 64 units.	269 epochs	Keras/Tensorflow	n/a	[46]
	Phage display CNN	Predict phage enrichment and generate novel CDRH3	96 847 sequences (largest dataset on github)	Ensemble of CNNs, largest with two convolutional layers and 18 706 parameters	20 epochs	Keras	link	[47]
	Image-based prediction	Distinguish between binding antibodies and lineages	24 953 models with calculated fingerprints from 308 EBOV and 54 HIV antibodies.	ResNet-50 [48]	Pre-trained model	Keras/Tensorflow	link	[33]
	Paratope and Epitope Prediction with graph Convolution Attention Network (PECAN)	Epitope and paratope prediction	162 structures for epitope prediction and 460 for paratope prediction	Graph Convolutional Attention Network	Up to 250 epochs, batch size of 32 (multiple parameters tested)	Tensorflow	link	[20]
	DLAB	Sorting of protein docking poses	759 Antibody– antigen complexes	Convolutional Neural Network	n/a	PyTorch	link	[32]
Embeddings/Language Methods	immune2vec	Embed CDRH3 into 100 dimensions using skip-gram	15,63 million sequences	Two dense layers	n/a	Gensim	link	[49]
	ProtVec CDRH3	Embed CDRH3 sequences to predict COVID-19 status	COVID-119 data from OAS	Based on ProtVec from Harvard DataVerse [50] and SVM	Reused previous model.	Reused previous model	link	[51]^*^
	AntiBerty	Masked language modeling, paratope prediction	558 milion sequences	BERT transformer encoder model, 8 layers, 26 M trainable parameters.	8 epochs, 10 days on four NVIDIA A100 GPUs	PyTorch	n/a	[39]^*^
	AntiBerta	Masked language modeling, paratope prediction	57 million sequences	Antibody-specific Bi-directional Encoder Representation from Transformers, 86 m parameters	12-layer transformer model that is pre-trained on 57 M human BCR sequences, 3 epochs, batch size of 96 across 8 NVIDIA V100 GPUs	PyTorch	n/a	[25]
	AbLang	Masked language modeling, reconstruct erroneous sequences	14 milion heavy chains, 200 000 light chains training. Evaluation sets of 100 k, 50 k for heavy lights respectively.	Based on RoBERTA from HuggingFace. 12 layers.	20 epochs for heavy chains, batch 8192, light chains 40 epochs 4096 batch size	PyTorch	link	[52]^*^
Generative methods/antibody design	Mouse VAE	Model latent space of CDR triples of antigen challenged mice	243 374 sequences.	VAE with encoder and decoder each having two dense layers (256 512 units each)	200 Epochs on a single GPU from the ETH cluster.	Tensorflow	n/a (available after peer review)	[53]^*^
	Developability-controlled GAN	Learn latent representation of human sequences and bias it towards biophysical properties	400 000 sequences	Generative Adversarial Network, (single chain) seven layers consisting of 2D convolution and dense layers.	500 epochs, batch size of 128	Keras/Tensorflow	n/a	[35]^*^
	Nanobody generation	Autoregression on nanobody sequences to generate novel CDRH3	1.2 milion sequences	ResNet with nine blocks with six dilated convolutional layers.	250 000 updates, batch size of 30.	Tensorflow/PyTorch	link	[17]
	*In silico* LSTM	*In silico* proof-of-principle of virtually unconstrained antigen-specific antibody sequence generation	70 000 murine CDR3 sequences	1024 LSTM with embedding layer and dense output layer.	20 epochs, batch size 64	Tensorflow	link	[54]
	Immunoglobulin Language Model (IgLM)	Masked language modeling, generate synthetic libraries of antibodies by solving masked language model	558 milion sequences	Transformer decoder architecture based on the GPT-2 model with 512 embeddings, 12 milion parameters	batch size of 512 and 2 gradient accumulation steps using DeepSpeed, 3 days when distributed across 4 NVIDIA A100 GPUs	GPT-2 from HuggingFace	n/a	[55]^*^
	IG-VAE	Immunoglobulins structure generation	10 768 immunoglobulins structures (including 4154 non-sequence-redundant structures)- set covers almost 100% of the antibody structure database (AbDb); Tested on 5000 structures from the latent space of the Ig-VAE	VAE	n/a	PyTorch	n/a	[34]^*^
	Generative method Benchmarking: (AR) the sequence-based autoregressive generative model, geometric vector perceptron (GVP) the precise structure-based graph neural network and (Fold2Seq) fold-based generative model	Antibody CDR regions design based on portion of sequence or structure.	Sequences from natural llama nanobody repertoire	AR- Autoregressive Causal Dilated Convolutions; GVP -based Encoder-Decoder GNN; Fold2Seq- Encoder-Decoder Transformer	n/a	n/a	n/a	[56]^*^
	GNN-based generation	CDRs sequence and 3D structure design	~5000 structures. For CDR-H1, the train/validation/test size is 4050, 359 and 326. For CDR-H2, the train/validation/test size is 3876, 483 and 376. For CDRH3, the train/validation/test size is 3896, 403 and 437.	Message passing network (MPN): Iterative Refinement Graph Neural Network (RefineGNN)	batch size of 16, dropout of 0.2 and learning rate of 0.0005	n/a	n/a	[23]^*^
	AntBO	CDRH3 region design		Bayesian Optimization and GP	87 cores 12 GB GPU memory	GPyTorch, Botorch	n/a	[57]^*^

For each method, we present the basic reported parameters used for training the network and approximate input. Wherever available, we report the architecture and libraries used to offer a point of reference for the currently used techniques in the field. Some methods (e.g. hu-Mab or mmCSM-AB) were not deep-learning-based, though they are included here for completeness. The non-peer-reviewed Biorxiv/Arxiv papers are indicated by ‘^*^’ in the Paper column.

DeepH3 is based on RaptorX [70] and used for *de novo* CDRH3 prediction. It is a deep residual network that given a one-hot encoding predicts the inter-residue distances and orientations into a discrete set of bins used to score poses generated by RosettaAntibody [71]. The training dataset consisted of records from SAbDab [72] with thresholds of 99% sequence identity and 3.0 Å resolution. The benchmark dataset consisted of 49 Fv structures selected from the PyIgClassify database [73], based on their quality, with CDRH3 loop of lengths between 9 and 20 residues. This method achieves accuracies in the region of 2 Å, rather than 4 Å in trRosetta [74] (which was designed for general protein structure prediction). This work showed that the distributions of orientation angles act as better discriminators than distance distributions alone. The latter were the hallmark of many previous methods in general protein structure prediction [10, 64, 70].

An alternative architecture for CDR (all loops) prediction in the form of E(G)NNs was proposed in ABlooper. Input data from SAbDab are encoded into 41-dimensional vectors with amino acid type, the atom type and which loop the residue belongs to. Additionally, sinusoidal positional embeddings are given to each residue describing how close it is to the anchors. Data from SAbDab were used to train five different E(G)NN networks, each with four layers. The agreement of the five networks on the generated coordinates is taken as the prediction confidence. An advantage of its method is its speed since it does not rely on other structure generation algorithms and can produce coordinates for thousands of structures within seconds.

Though predicting CDRH3 loops is the most challenging task, reconstructing the entire antibody variable region is the overarching goal. DeepAb is built on top of the DeepH3 method, but it is designed to predict the whole variable regions. The method consists of two main stages: a deep residual convolutional network predicting bins of distances and orientation angles and a Rosetta-based protocol for structure realization of the predicted distance and angle constraints. Additionally to the structural information fed as input, the network incorporates a bidirectional-LSTM network trained on a set of 118 386 paired heavy and light chain sequences from observed antibody space (OAS) [8] to teach the network the general features of antibody sequence space. Such feature extraction allowed for implicitly capturing certain structural properties, such as recreating the PyIgClassify annotations [73]. Furthermore, the network tracks residues contributing to coordinate/angle prediction of each other via an attention mechanism. The network primarily attends to residues surrounding each loop of interest, with the distinction that CDRH3 predictions draw from a broader set of dependencies across the heavy and light chains.

Though methods such as DeepAb demonstrate the power of machine learning techniques in antibody structural predictions, they can be hindered by slower methods required to generate coordinates. This problem was addressed by NanoNet [18], which was designed to predict structures of single antibody chains. Originally designed for the prediction of single-chain nanobodies, it is a residual convolutional network that produces predicted coordinates as output when given a variable region sequence. NanoNet aligns the input structures, creating a single frame of reference regarding which the predictions are being made. Since the structure realization is accomplished within a single network, it can produce thousands of structures in a matter of seconds.

The methods discussed above provide a tangible performance increase in terms of the most challenging problem, the CDRH3 prediction being achieved now in the region of 2 Å rather than 3-4 Å beforehand [75]. The predictions are approaching sufficient levels to be reliably used as substitutes for crystal structures, specifically providing models at speed and scale [76] necessary to tackle the antibody-binding problem for the ultimate prize of virtual antibody screening.

## Embedding the ab-ag space: prediction of antibody–antigen binding

Three-dimensional structures of antibodies and antigens are important determinants of antibody–antigen interactions. Typically, the development of novel binders was confined to animal immunizations of phage-display methods. Methods used to analyze antibody–antigen interactions were previously categorized into paratope [43, 77, 78], epitope [79–82], or docking [83–85] reviewed previously [7]. Although certain previous methods, such as Parapred [43], pioneered the use of deep learning in predicting antibody–antigen interfaces (paratopes specifically), the current combination of next-generation sequencing and machine learning methods accounts for novel applications going beyond the confines of the three categories (Table 1).

Deep learning methods are combined with high-throughput sequencing to improve the predictions obtained from display technologies. In order to get high-affinity binders, one needs to perform several costly and time-consuming panning rounds. To address this, Saka and colleagues employed binders from their panning experiments (against hapten kynurenine) to train an LSTM model using 959 heavy sequences [46]. This model provided a basis for capturing the features of their binding antibodies to sample novel binders. They used their model to generate novel sequences by sampling amino acids and feeding these to the model to obtain consecutive amino acids in sequence. At the softmax layer (that gives a likelihood of each amino acid at a position), they added a temperature factor that introduced more randomness. Following such a generative strategy, they removed sequences with amino acids in positions not seen in the training set. They hypothesized that the generated antibodies could be better binders; indeed, the best one achieved a significant improvement concerning the parental sequences.

Another application to improve the phage display technology was proposed by Liu et al. They predicted phage enrichment (better binders) against ranibizumab, bevacizumab, etanercept and trastuzumab based on CDRH3 sequences [47]. They ran three rounds of panning against ranibizumab, with predictions on the enrichment of round 2 to round 3. They employed a CNN to predict this property, the largest of which was a 2-layer CNN. They trained ensemble classifiers from the trained models that outperformed the individual models on held-out data, showing that the model generalizes to unseen data. The authors also trained separate ensembles on anti-bevacizumab, anti-etanercept and anti-trastuzumab antibodies. They removed anti-bevacizumab predictions with a higher score for etanercept and trastuzumab, avoiding 75% of such non-specific predictions. To generate new sequences, they used a seed sequence that they optimized using their ensemble. They keep the same network parameters but use the back-propagation to project a new version of the input sequence—if the score is not improved within 10 iterations. They compared the results of their generated sequences. Two improved binders (1.899 and 2.888 log_10_ round 1- to round 2 enrichment) were only two mutations away from the seeds. However, exploring all such 2-point mutations in 6566 used seeds would translate to 2.193 × 10^8^ sequences making *in silico* exploration of this space much faster and more economical.

Identifying an antibody binder needs to be coupled with selecting those exhibiting favorable developability properties. This problem was tackled by Mason et al. [86], who combined experimental data generation with subsequent neural network binding training and developability filtering. They generated 11 300 and 27 539 binders and non-binders towards Her2 based on trastuzumab. They benchmarked several neural network architectures on the problem of predicting the binding probability. Out of several standard architectures such as plain Artificial Neural Network, LSTM, Random Forest, support vector machine (SVM), the CNN achieved the best discriminating performance and was chosen as the standard model. The objective function to predict binders was employed to select putative binders from computationally generated sequences (based on the Deep Mutational Scanning profile of trastuzumab CDRH3). The authors generated 7.2 × 10^7^ sequence variants and used their CNN predictor to select a set of sequences that were predicted to bind (*P* > 0.7) and not (*P* < 0.1), stipulating that they need to have at least a Levenshtein distance of 5 from the originator trastuzumab. The authors confirmed experimentally that 30 of the predicted binders and 11/12 of non-binders did not bind. One of the binders experienced an almost 4-fold increase in binding affinity concerning the original trastuzumab. The authors calculated the Fvcsp [87], Camsol score [88] and NetMHC2PAN [89] to filter the set of predicted binders. They experimentally validated 55 variants to demonstrate that one of them had a comparable expression profile, better solubility and a putatively better immunogenicity profile. This showed how one could combine experimental screening with machine learning binding models to select variants with favorable therapeutic properties.

Even if improving experimental screening via machine learning undoubtedly facilitates the therapeutic discovery process, it still falls short of the ultimate prize, which is the generation of antibodies purely *in silico*. For this, one requires an objective function to determine whether an arbitrary antibody–antigen complex could interact. This problem was tackled by Pittala and Bailey-Kellogg using neural graph networks [20]. Each protein structure is represented as a graph, with nodes for the amino acid residues and edges between residues with Cβ-Cβ distance <10 Å (with Cα for Gly). Each residue is associated with the one-hot encoding of the amino acid type, surface accessibility, psi-blast conservation profile and local (<8 Å) amino acid context profile. When given graphs for the two input proteins, the network learns the probability of a given residue being part of the recognition interface. Their method performs better than previous methods, namely EpiPred [80] and Discotope [82]. One of the key improvements of the method is the attention layer, which indicates the scores contributing to the final predictions for each residue.

Another application of developing a predictor of antibody–antigen interactions was proposed by Ripoll *et al*. [33]. The authors aimed to predict structural interfaces by using image recognition paradigms. They assumed that distinct antibodies targeting the same epitope need to share some features that are specific to the particular antigenic configuration. Therefore, they created fingerprints of the antibody binding site and projected them onto a plane for image recognition, labeled with a particular epitope. On the basis of ResNet-50, they trained a deep convolutional network to predict Ebola and HIV epitopes. They identified datasets of anti-Ebola and anti-HIV antibodies and modeled them using RosettaAntibody [27] to obtain the structural fingerprints. The anti-HIV dataset consisted of 7310 fingerprint models from 53 antibodies, with the Anti-Ebola dataset comprising 17 643 models from 308 antibodies. They employed the classifier to distinguish the fingerprints from a single lineage from a pool of unrelated fingerprints.

The ultimate goal of antibody–antigen interaction prediction is enabling researchers to employ a large volume of NGS data to mine for novel binders, termed ‘virtual screening’. Such virtual screening attempts in the field of small molecules are often combined with large-scale docking [90]. Deep learning models are increasingly used to score the different docking poses for protein–protein functions in general [91–93], with antibody–antigen docking treated as a separate case [85, 94] (reviewed recently [95]). Docking was employed recently by deep learning for antibodies (DLAB) to address virtual screening by rescoring ZDOCK [96] poses in an antibody-specific fashion. The network used to re-score docking poses was a deep convolutional network that classified the poses into a bucket, indicating an interval of the fraction of native reconstructed contacts (*f*_nat_). Though the overall prediction of docking scoring was improved, the method did not achieve strong discrimination between binders and non-binders.

All the approaches above use novel datasets and machine learning models to predict antibody–antigen interactions. However, none provides the ultimate general ‘objective function’. To address the issue of ‘learnability’ of the Ab-Ag recognition, Akbar et al. tested this concept *in silico* by simulated Ab–Ag binding data [54]. They trained the LSTM-RNN on CDRH3 that were apriori computationally associated with developability data [13]. The network can generate sequences that exceed the developability parameters of the sequences used for training the network. It is demonstrated that the network can also generate specific binders against HER2 by giving it binders against this target to train on. This shows that antibody properties are, in principle, trainable on a multitude of its modalities, binding and developability.

## Developability—deimmunization using large-scale NGS data and machine learning

An antibody binder towards a therapeutic target should meet a range of biophysical features, termed collectively as manufacturability/developability [97]. Previous approaches to tackle this issue employed statistical models [59] or non-deep learning approaches such as random forests [98] that were reviewed in-depth elsewhere [99]. The increasing availability of NGS data and developments in machine learning have spurred progress in a specific branch of developability, namely deimmunization.

Natural B-cell repertoires of mice, sometimes engineered human germline repertoire, often serve as the source of therapeutic antibodies. However, animal antibodies administered to humans may induce an immune response that can neutralize the therapeutic effect of the antibody. To avoid this, antibodies must be engineered to resemble human antibodies without loss of activity in a humanization process [100].

Traditionally, humanization was approached using frequency-based methods quantifying the similarity of the animal sequence to human ones (e.g. T20 [101] or humanness scores [102]). Such approaches, however, were based on a small number of sequences (in thousands), giving limited ability to learn the correlation between different residues. The availability of NGS increased the antibody sequence samples from the order of magnitude thousands to millions. Improved positional frequencies were created based on such data [103, 104].

Even if enriched by NGS data, positional profiles lack the positional correlation granularity. To quantify possible positional correlations, an multivariate gaussian (MG) statistical score was developed based on the OAS data [105]. This was later expanded to an LSTM model by Wollacott [31]. Both models focused on predicting what constitutes the human sequence, introducing the necessary element of correlations between positions. Crucially, the authors of MG compared their score to the immunogenicity of therapeutic sequences, though it resulted in a weak correlation (*r*^2^ = 0.18), indicating that sequence identities alone might not encode the immunogenicity information. This observation is consistent with the industry experience on the origins of immunogenicity. Immunogenicity towards biotherapeutic drugs is often observed in clinical trials via the generation of Anti-Drug Antibodies (ADAs) by the patients receiving immunotherapy. The origin of immunogenicity in patients is multifactorial, with factors related to drug product quality (e.g. formulation, presence of aggregates in the product or aggregation of the product *in vivo* upon administration), patient’s disease history, and their genetic background playing crucial roles, along with humanness of the antibody sequence [106–108].

Though Wollacott and colleagues employed NGS data, they only used a relatively small fraction of OAS, namely 400 000 sequences. By contrast, the authors of hu-mab [42] used a far more extensive dataset (Table 1), also drawn from OAS. Their method was not based on a deep learning framework, but on a random forest model trained to distinguish human and non-human sequences of a specific V gene type from ones originating from other species. Hu-mab correctly discriminated between human and other animal sequences in both validation and test sets, with slightly worse performance on the light chain, which might be caused by the greater amount of negative training data available for the VH models than VL models, but also because of smaller variability of light chains both in terms of isotypes and CDRs. The previous LSTM model [31] was not entirely capable of discriminating between human and other animal sequences, which can be because LSTM models were only trained on sequences from a single species.

Another computer method that intends to accelerate the process of humanization is BioPhi [24], an antibody design interface with automated methods that capture the diversity of natural human antibody repertoires. By combining adaptive immune repertoire sequencing and antibody engineering, BioPhi integrates two data-driven methods—novel humanization (Sapiens) and humanness evaluation methods (OASis). Sapiens is a deep learning humanization method based on masked language modeling (MLM) trained with human variable region antibody sequences from the OAS. Sapiens is trained to recognize and repair masked or mutated positions in unaligned amino acid sequences. OASis is a humanness metric based on peptide search in the OAS. OASis evaluates the humanness of an antibody sequence by dividing it into all overlapping 9-mer peptides (inspired by human string content [109]) and then comparing them against the OAS database to predict their universality across the human population. Based on an *in silico* humanization benchmark of 177 antibodies, this software offers mutational choices similar to ones achieved by experimental humanization methods. The chief advantage of BioPhi is its attention layer and granularity that allows the user to examine the residue dependencies and mutational effect on the score. By drawing from language models, BioPhi pioneers a new trend in antibodies where such methods are used not only to provide solutions to established problems such as humanization but also to open new areas of research altogether.

## New opportunities in computational antibody design owing to deep learning

Machine learning methods fundamentally learn a latent embedding of the input space. This can be interpreted as a vector space where features of the input instances and associations between them are implicitly accounted for. In the antibody world, this can mean sequence-similar antibodies, or even more abstractly, distinct paratopes sharing identical/similar antigens. With a large amount of NGS data, employing such methods—often drawn from natural language processing (NLP)—opens opportunities for encoding the antibody sequence space with learned embeddings and employing it for transfer learning (e.g. paratope prediction). More importantly, it allows for a radical paradigm shift in antibody design as novel sequences with pre-defined properties can be ‘sampled’ from such learned latent representations, or embeddings.

## Embedding the antibody sequence space: applications of natural language-processing techniques in antibodies

An emerging strand in antibody sequence analysis is employing NLP to develop embeddings of antibodies (Table 1). Protein and nucleic acid sequences can be simplified to a textual representation allowing embedding these in vector space [50, 110]. The purpose of such an operation is to transform sequences into a vectorized representation [50] that implicitly accounts for intrinsic biophysical properties (e.g. function-similar proteins should be closer to each other in vector space).

Antibodies are well suited for NLP applications as they are proteins characterized by great molecular diversity, estimated to be as many as 10^18^ unique molecules [111]. Therefore, it is plausible to draw parallels between words, sentences, documents, amino acid k-mers, CDRs, frameworks and antibody repertoires. With multiple NGS datasets annotated with disease states available now, the application of NLP methods in antibodies holds the potential to encode the antibody space, revealing novel insights into the biology of immunoglobulins.

NLP’s word2vec provides an embedding for a natural language word. Here, words semantically related by context, are also close in vector space. Word2vec-inspired unsupervised learning was used by Protvec for antibody data [51]. Protvec started with the original sequence split into three separate lists of non-overlapping 3-mers, which are trained on 546 790 sequences from Swiss-Prot [112]. The vector representations were then summed into a 100-dimensional vector representing a single protein sequence. In the specific case of antibodies, the authors encoded the CDRH3 sequences to offer an embedding for a single immunoglobulin sequence using Immunoglobulin G heavy chain (IGHG) sequences from OAS [8]. The Protvec embeddings were used for classifying and tracking b-cell receptor (BCR) repertoires of COVID-19 patients and healthy individuals. Authors encoded the entire repertoires by adding vectors from 100 most common sequences in a repertoire that separated into clusters of either healthy patients or those with an ongoing COVID-19 infection.

Nevertheless, Protvec was only used to produce the embedding without explicitly training the network. By contrast, Immune2vec used the word2vec framework to adapt it to antibody sequence embedding [49]. Here, the CDRH3s are tokenized into non-overlapping 3-grams (three consecutive amino acids). On this basis, the word2vec model predicts the surroundings of a given word based on n-gram sequences without knowing the labels (with window size set to 25). Such unsupervised learning captures some of the biochemical and biophysical properties of the 3-grams, implicitly classifying the sequences according to their corresponding Immunoglobulin heavy chain variable region (IGHV) families. The embedding was further applied to classify samples from hepatitis C virus (HCV)-positive patients. Repertoire level representation was achieved by clustering the 100-dimensional representations, using random forest to identify the most relevant features, followed by logistic regression achieving close to 90% prediction accuracy.

Previous methods attempted skip-gram modeling to predict the rest of the sequence based on a stretch of amino acids. This is related to another notion in language modeling, specifically MLM. Here one obscures (masks) part of the text and attempts to recreate it based on the learned context. There are currently three methods that were proposed for this problem, AntiBERTa [25], AntiBERTy [39] and AbLang [52].

AntiBERTa [25] is a Bidirectional Encoder Representations from Transformers-based transformer with 12 layers and a total of 86 million parameters. For training, random residues are masked and the task is to predict these. The latent representation reflects multiple features of antibody function, such as correspondence with ADA scores and discrepancy from the germline. The pre-trained AntiBERTa was used for binary prediction of whether a residue is part of the paratope or not. Compared to Parapred and pro-ABC, AntiBERTa achieves the highest precision of the methods at 74%. The ability to predict paratope positions outside the CDRs was a significant advantage. This method makes particular use of the attention mechanisms that can reveal the context of the entire sequence that influences the predicted position. For instance, AntiBerta does not focus on the invariant disulfide bridge in antibodies between 23–104.

With a convergent name, AntiBERTy [39] is also based on the transformer model of BERT architecture, obtained from HuggingFace, attempting the MLM task. Using the embeddings, they construct k-nearest neighbor graphs for individuals producing anti-HIV antibodies. Without being specifically trained to do so, after visualizing the embeddings from anti-HIV producing individuals, one could note the trajectories of differentiation from the germline akin to the typical affinity maturation process. They defined the problem of identifying paratopes as identifying highly redundant features within the repertoire. On a set of antibodies from anti-HIV-producing individuals and compared against known anti-HIV structures, they found binding consistencies indicating orthogonal paratope prediction capacity to that of AntiBERTa.

AbLang is a transformer adapted from HuggingFace (specifically, Roberta [113]) and trained on OAS data. Its main application is filling in missing portions of sequences lost in the high throughput sequencing process. Following typical MLM protocols, several residues are chosen to be masked, tasking the predictor with inferring them. Each residue is encoded into 768-dimensional vectors. AbLang provides encodings for all residues or entire sequences (mean residue encodings). AbLang encodings implicitly group the vectors by V-genes. When compared to the task of filling missing N-terminal residues, protein-based model evolutionary scale modeling 1b (ESM-1b) [114] performs worse than AbLang and copying germlines. Authors note, however, that AbLang is comparable to just employing germline information.

One of the main assets of the language models is learning the latent space of antibodies, which implicitly accounts for certain features. This can be treated as a space from which one can sample novel antibodies, changing the paradigm of computational antibody design.

## Sampling the embedded antibody space: generative methods for novel antibody sequences *in silico*

One of the biggest challenges in therapeutic antibody discovery is finding novel sequences. Antibody sequence space is estimated to cover up to 10^18^ unique molecules [111, 115]. One of the traditional approaches to finding a binder was to create a sample of this space in the form of phage display libraries. These libraries can reach a diversity of 10^11^ possible molecules, which is a small sample of the total possible space and does not guarantee the reproduction of naturally functional molecules [116]. Animal immunization provides access to the entirety of the animal repertoire. However, this is a burdensome approach where molecules still need to be engineered for favorable developability properties [117]. Therefore antibody engineers are faced with the problem of how to traverse the antibody sequence space, only enumerating the antibodies that can be functional.

Previous computational antibody design methods operated based on enumerating sequences, mutations, or structural variants and then attempting binding prediction [118–120]. Such methods emulated physics to sample novel conformations reflective of fundamental rules of nature but might not explicitly account for strategic statistical biases in biologically and therapeutically relevant antibody space. From a statistical standpoint, despite great diversity, the antibody sequence–structural space does not follow uniformly random patterns. Convergences can be found in the identical CDRH3 sequences developed by different individuals responding to the same pathogens [121]. Despite following different ‘development pathways’, multiple therapeutic CDRH3 sequences can be found in naturally-sourced repertoires [122, 123]. Antibody structure space (short of CDRH3) appears to be particularly constrained to a certain number of folds [76].

**Figure 3:**
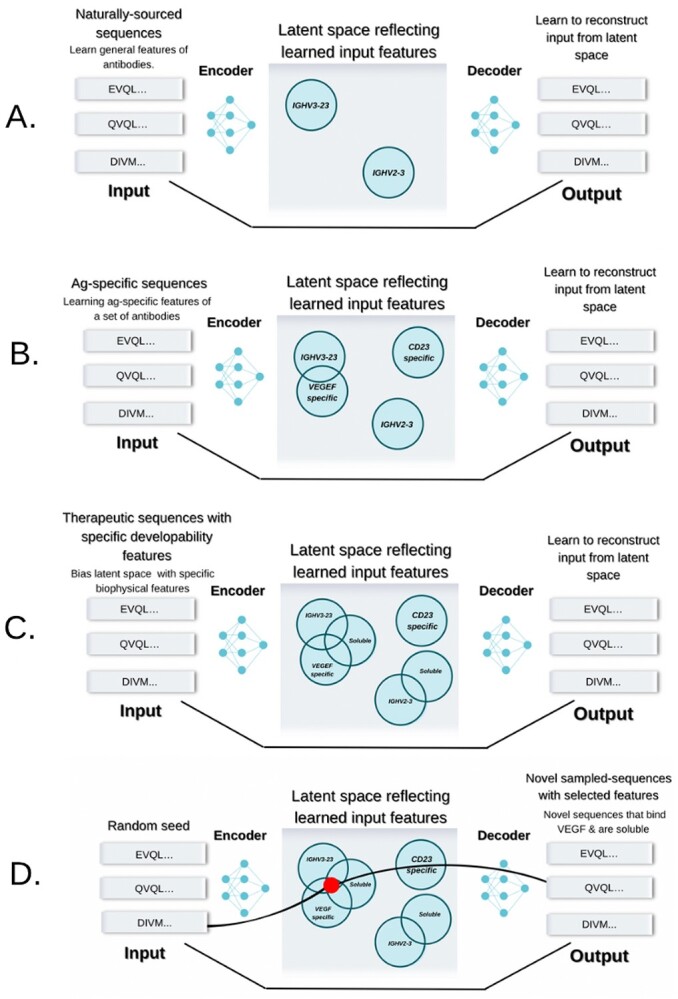
Generative methods for computational antibody design. (**A**) Millions of natural NGS sequences can be used to learn the general features of the antibody sequences, such as positional frequencies, amino acid dependencies and gene groupings. (**B**) Feeding antigen-specific sequences, one can bias the distribution to learn the features of sequences specific to a given antigen. (**C**) Sequences that have known favorable biophysical properties (e.g. solubility, low immunogenicity) can be used to bias the latent space towards such features. (**D**) One can use the latent space to randomly sample points from it in a directed fashion that complies with certain specifications, such as specificity and biophysical properties.

Revealing the biological contours of the antibody molecular space can be addressed by a novel field within computational antibody discovery, namely Generative Networks [56] (Table 1). A neural network by design attempts to learn the latent distribution of the input space (Figure 3). Therefore, one can also employ the learned latent space to sample from it—sequences or structures. There exist certain limitations, though, as to be sampled reliably, the space should ideally be isomorphic and continuous. The alternative is a disorderly latent space which though encoding the input in efficient representation, does not allow to sample reasonably. Architectures such as VAEs or Generative Adversarial Networks address this problem by learning to reconstruct input and forcing the latent space to be ‘well-behaved’. Specifically, within the field of antibody design, such networks can be used to learn the latent space from input sequence [56] and bias it towards specific binders and developability properties (Figure 3).

As an example of such an approach, Friedensohn et al. immunized 45 mice with four antigens (OVA, HEL, BCP and RSV-F) and collected approximately 240 000 combinations of three CDRs in total (though antibodies were not sorted by antigen specificity) [53]. They assumed that the sequences in the latent space were generated using a Gaussian Mixture Model to train a VAE to capture the distribution of the training data. The VAE was tasked with placing one-hot encoded input CDR combinations into a cluster, the number of which is pre-defined. The groupings are imposed to reveal relationships between the sequences that would not be apparent from clonotyping. At around 2000 clusters and sequence dimensionality of 10, the reconstruction accuracy plateaued at 93%. The VAE captured obvious relationships such as reflecting closeness between CDRH3 lengths and variable gene segments being mapped to corresponding areas of the latent space. They used sequences placed in the same cluster as a confirmed RSV-F binder to check for shared specificity. All 12 sequences selected in this fashion bound the antigen and were sufficiently dissimilar so as not to be called convergent binders using traditional clonotype definitions. They further studied the sequences in the RSV-F binder cluster by sampling latent representations from these. A total of 5005 novel sequences were generated in this way, 96 were checked experimentally, 71 of which (74%) were binders.

Sequences can also be generated by predicting the consecutive residues in the sequence, called ‘autoregression’ [17]. An autoregressive model was proposed by Shin et al. where a combination of ResNet with dilated convolutions was used to model the following elements in the sequence of amino acids (WaveNet [124]). They used 1.2 milion nanobody sequences to learn the distribution of amino acids in these sequences. It was assumed that such natural sequences would be associated with favorable biophysical properties, such as stability. Using germline CDR1 and CDR2 sequences as starting points, they generated new CDRH3 sequences one amino acid at a time and rejected those that do not fit the constraints of nanobodies (e.g. ending in beta-strand). In total, they generated approximately 3.7 m sequences, and of these, they got 185 836 CDRH3s as seeds for the experimental library generation. The nanobodies coming from this library were confirmed to have better expression. They further showed that this library contains weak/moderate binders to human serum albumin, suggesting a low probability of non-specific interactions.

Another generative method, Immunoglobulin Language Model (IgLM) addresses the issue of unidirectional autoregressive methods, where the prediction of the following amino acids is only dependent on the previous ones. Here the authors perform predictions to redesign parts of the sequence, taking the entire context into account [125]. For sequence generation, they use the Generative Pre-trained Transformer (GPT)-2 architecture from the HuggingFace repository. For training, they use 558 milion sequences from OAS. As expected, prediction performs the worst on the CDRH3 region and the best on the framework regions while learning residue embeddings that capture their physicochemical properties. To generate new sequences, they start with the beginning residues of framework 1 (e.g. EVQ) and predict the following (masked) residues. They introduce the temperature factor that increases the diversity in predictions to introduce the randomness of generated sequences. On the basis of the predictor, a library of CDRH3 sequences is generated. They computationally analyze the library using tools CamSol and spatial aggregation propensity (SAP), showing that the *in silico* generated sequences have better (predicted) properties than a random mutagenesis library or grafting existing CDRs onto the framework.

Though previous methods either assumed better developability properties based on input data (e.g. better thermostability [17]) or performed filtering based on predicted properties [86], they were not specifically biasing the latent space for that task. This was addressed by Amimeur et al., who used 400 000 sequences from OAS as a base model of antibody amino acid sequences [35]. Such a base model was used for transfer learning, biasing the predictions using smaller datasets with known developability properties. They used transfer learning to bias towards: (i) shorter CDR lengths, (ii) major histocompatibility complex (MHC) II binding prediction for immunogenicity, (iii) isoelectric point and (iv) lower negatively charged patch on the molecular surface. Using the sampled sequences, they experimentally produced full-length antibodies and experimentally checked their behavior across four metrics: differential scanning fluorimetry (DSF, thermostability), self-interaction nanoparticle spectroscopy (self-interaction), polyethylene glycol (PEG) solubility and size-exclusion chromatography (solubility), showing that their molecules indeed fall within acceptable ranges for these assays.

All the previous pieces of work focused on sequence generation, disregarding the structure which actually bestows the binding specificity. This was addressed by the authors of IG-VAE, who used antibody structure data from the database ABDB [126] to generate a VAE learning the latent structure representation of antibody molecules [34]. IG-VAE is a backbone generation algorithm that does not generate the associated sequence. The VAE model reconstruction loss was composed of the torsion and distance, trying to optimize both, though authors note that in the beginning that they had to up-weigh the torsion loss. They tested the ability of the VAE to reconstruct the structures by generating 500 structures and comparing their geometries to a non-redundant set of 500 real structures. The dataset did not naively recapitulate the training set as the authors discover a set of novel loop shapes that still have plausible bond geometries. To test whether the novel backbones could be associated with a sequence, the authors used Rosetta FastDesign, which puts an amino acid sequence on a backbone. They demonstrated a proof of concept of how one could employ the method for designing molecules by designing backbones targeting the ACE2 epitope of severe acute respiratory syndrome coronavirus type 2 (SARS-CoV-2) receptor binding domain (RBD). They generated 5000 backbones and performed docking using PatchDock [127]. Two decoys with favorable complementarity were confirmed as low energy according to Rosetta Energy Units though not experimentally confirmed. Finally, the authors showed that it is possible to specify constraints to the network such as distance constraints for a loop, antigen positioning and complementarity, showing the potential to control for a set of desired properties.

Altogether, the generative methods provide a novel paradigm in computational antibody design that employs learned representations of structure and sequence space. To fully deliver on the promise of *in silico* antibody generation, these methods must be made sufficiently generalizable without the initial experimental data generation steps.

## Discussion

The steady development of data resources and associated computational methods addressing therapeutic antibody design increases the role of *in silico* methods in antibody discovery [7, 99, 128]. One of the seminal works on computational antibody design was the framework of Lippow in 2007 [118]. It was molecular mechanics-based (CHARMM forcefield [129]), trying to capture the physics of studied molecules that set the tone for antibody design for the next decade [7].

By contrast, machine learning methods do not aim to reproduce physical phenomena but rather to distribute observed data. Recent advances in machine learning, culminating in a dramatic performance by AlphaFold, will keep inspiring similar work in the context of antibodies. Attempts at expanding AlphaFold work in the antibody sphere have already been made beyond structure prediction to molecular complex prediction [130]. Structural modeling of the proteome in AlphaFoldDB also describes the structural molecular space that is potentially druggable [131]. Seeing how AlphaFoldDB solved an age-old problem in bioinformatics using publicly available data, it is encouraging to think that a similar feat of clever data re-use and model development could be reproduced in certain spheres of computational antibody design.

Machine learning approaches are already making their mark on antibody bioinformatics, such as structure prediction. They streamline the existing discovery methods, such as identifying antigen-specific sequences that have long been dominated by clonotype methods that rely on sharing germlines and high (>80%) CDRH3 sequence identity. Using paratope predictions to select convergent binders (called ‘paratyping’ [132]) perhaps was not outright better than clonotyping. However, it provided alternative identifications to clonotypes. The VAE [53], introduced by Friedensohn, was better at identifying convergent sequences than clonotyping by grouping antigen-specific sequences across multiple features. However, further investigations on ground truth data are needed for an unbiased ranking of ML-based antibody bioinformatics approaches [13, 54].

The contribution of deep learning methods to antibody discovery is not constrained to streamlining existing methods—it is redefining how the discovery process is approached. Generative methods offer a tangible way to encode the natural and therapeutic features of antibodies to sample novel sequences purely *in silico*. Though existing methods need to be fully decoupled from experimental methods to deliver on their potential fully, they do set the tone for the future.

Entirely *in silico* antibody generation is within reach, and when it is achieved, it will open a new chapter in therapy development. Rapid identification of antibodies combating emerging viral diseases is necessary to use the full potential of these molecules [133]. This would also open the opportunity in precision medicine. Currently, a single antibody sequence is used to treat multiple patients. The caveat of this approach is reflected in highly varied patient-drug responses. Tailoring a drug to a patient in a highly complex system is currently out of reach because of time and resource limitations. However, this goal will become achievable if antibody discovery speeds up thanks to *in silico* methods.

On a more immediate note, *in silico* antibody discovery will dramatically reduce the time and costs required for drug discovery projects. For example, the current timeline required from the definition of a novel therapeutic concept to the start of development is approximately 2–3 years. By combining computational technologies of drug discovery with affinity maturation and developability assessments, it may be feasible to reduce this timeline to <6 months, including the time required for experimental validation and characterization of the lead molecules. The enablement of computational technologies for biologic drug discovery will also help overcome the hurdles associated with poor solubility and conformational instabilities of the target molecules such as membrane proteins. The costs associated with drug discovery and development shall also be reduced because computation does not require any material.

Any future applications will crucially depend on data curation and method development. There is an ever-growing ecosystem of free federated data covering a broad spectrum of antibody data types [1, 8, 72, 123, 126, 134–137]. The availability of antibody-specific antibody models is also encouraging, as many methods in Table 1 make their predictors available. Tying data and models together, no matter how sound the benchmarking and reproducibility, is still challenging even when all data is available. This is due to diverse setups in the multitude of available parameters. Such issues are addressed by frameworks that perform data collation and acquisition [8, 134] and formalize the machine learning pipelines such as ImmuneML [138].

Though we expound on the benefits of deep learning, this approach should not be treated as a panacea and its drawbacks should be acknowledged as well. Though deep learning offers a practical solution to many data-driven problems, these architectures can lack interpretation. For instance, in a specific case of AlphaFold, the method might not shed light on the underlying physical processes [139]. Side-stepping biological interpretation can be convenient for practical purposes but not desired in general as such fundamental understanding is important for developing safe drugs. Furthermore, in many applications, much data is needed. This is not an issue in some areas of antibody discovery, such as embedding antibody sequences which can rely on millions of data points from NGS. Predicting solubility and immunogenicity is more challenging as data paucity exists. Such considerations are pertinent regarding the dangers of misuse of models, as such models carry the danger of overfitting data if not used properly. Despite the drawbacks, deep learning provides a novel approach that should be espoused by the pharmaceutical industry to streamline drug development.

Furthermore, the advent of deep learning did not replace all the other computational approaches altogether (see recent review here [99]). Non-machine learning statistical methods in the therapeutic antibody sphere continue to be developed [123, 140, 141]. Learnings from antibodies are being transferred to their sisterly format such as nanobodies [18, 135, 141]. Of note, synergies between existing data sources provide novel findings, such as employing structural information to annotate large NGS datasets [76, 95, 142–144]. The increasing momentum of computational methods is therefore encouraging to speed up the development of therapeutics by the biotechnology industry.

Within biopharma and biotechnology companies, there is also a critical need for embracing digital transformation by actively curating the data on discovery and development projects and using it to connect microscopic molecular properties with macroscopic experimental observations via a combination of machine learning and molecular simulation methods. Making this paradigm shift in how biological drug discovery and development projects are prosecuted will help realize the vision of Biopharmaceutical Informatics which calls for syncretic use of modern computational and experimental technologies to make biological drug discovery more efficient [7, 99, 145, 146].

In our review, we focused on providing a systematic overview of deep learning in the therapeutic antibody context that would help biopharma companies espouse these concepts for the benefit of faster and more efficient drug development. Deep learning methods have given new momentum to the computational antibody design field by showing a realistic path for future pipelines having artificial intelligence (AI)-designed antibodies. The accurate measure of the success shall be the translation of AI-enabled biotherapeutic drug discoveries into medicines available in the market after passing through all the challenges associated with drug production and clinical development. The realization of their promise shall require embracing a new ‘culture’ of computation by the industry. This requires therapeutic project execution and data generation that are intrinsically data and prediction driven.

**Key Points**

- Machine Learning methods in general and Deep Learning approaches in particular are incresingly being applied to analyzing and designing novel therapeutic antibodies.
- Use of Deep Learning not only provides improved solutions to existing problems in computational antibody design, such as structural modeling, but also opens new avenues such as language-inspired modeling.
- Generative Modeling applied to antibodies offers new opportunities of in-silico designing novel molecules with desired properties.
- Realisation of the full potential of Deep Learning methods in therapeutic antibody discovery would require a paradigm/cultural shift in the way novel biotherapeutics are discovered, by increased use of computational methods.

## Author Biographies

**Wiktoria Wilman**—Bioinformatician at NaturalAntibody, researching and developing antibody data analysis tools.

**Sonia Wróbel**—Senior product manager at NaturalAntibody, focusing on developing science-based products for antibody therapeutics design.

**Weronika Bielska**—Junior database specialist at NaturalAntibody and medical student at the Medical University of Lodz.

**Piotr Deszynski**—VP Engineering at NaturalAntibody, developing infrastructure for data acquisition and analytical stack for computational antibody characterization.

**Paweł Dudzic**—Senior data engineer at NaturalAntibody, focusing on data federation and machine learning pipelines.

**Igor Jaszczyszyn**—Junior database specialist at NaturalAntibody and medical student at the Warsaw Medical University.

**Jędrzej Kaniewski**—Data scientist at NaturalAntibody, interested in applying machine learning methods to problems in bioinformatics.

**Jakub Młokosiewicz**—Data scientist at NaturalAntibody, focusing on structural aspects of antibody characterization.

**Anahita Rouyan**—Consultant at NaturalAntibody, responsible for product and service marketing.

**Tadeusz Satława**—Senior data scientist at NaturalAntibody, focusing on language models in immunoinformatics.

**Sandeep Kumar**—Distinguished research fellow at Boehringer—Ingelheim Pharmaceutical Inc., USA. Sandeep has been advocating for the vision of biopharmaceutical informatics that calls for synergistic use of computational technologies, such as machine learning and digitalization of experimental data, to improve the productivity of biologic drug discovery and development projects.

**Victor Greiff**—Associate professor for Computational and Systems Immunology at the University of Oslo and Oslo University Hospital, focusing on systems strategies for antibody design.

**Konrad Krawczyk**—Founder of NaturalAntibody, focusing on the development of novel computational protocols to characterize antibody therapeutics.
